# Peroxisomal fatty acid oxidation-related signature for predicting prognosis and therapeutic response in low-grade glioma

**DOI:** 10.3389/fonc.2026.1897651

**Published:** 2026-08-13

**Authors:** Xiaobin Zhou, Hui Liang, Qinhong Huang

**Affiliations:** 1 Department of Neurosurgery, The First Affiliated Hospital of Shantou University Medical College, Shantou, Guangdong, China; 2 The National Key Clinical Specialty, The Engineering Technology Research Center of Education Ministry of China, Guangdong Provincial Key Laboratory On Brain Function Repair and Regeneration, Department of Neurosurgery, Zhujiang Hospital, Southern Medical University, Guangzhou, China

**Keywords:** biomarker, FAO, LGG, machine learning, peroxisome

## Abstract

**Introduction:**

Metabolic reprogramming is one of the hallmarks of cancer. Increasing evidence indicates that lipid metabolism reprogramming plays an important role in the development of tumors, especially glioma. Fatty acid oxidation (FAO) occurs in both mitochondria and peroxisomes. At present, studies of FAO have mainly focused on mitochondria, while research on peroxisomes remains limited.

**Methods:**

This study utilized bioinformatics methods and functional assays to explore the potential role of peroxisomal FAO in low-grade glioma (LGG).

**Results:**

As a result, we constructed a reliable peroxisomal FAO-related prognostic model for LGG based on different combinations of 10 well-performing machine learning algorithms. Gene set enrichment analysis (GSEA) and drug sensitivity prediction analyses suggested that the identified prognostic peroxisomal FAO-related genes may contribute to therapeutic resistance in LGG. Functional assays further demonstrated that phosphomannomutase 2 (PMM2) could be a potential therapeutic target for LGG. Downregulation of PMM2 not only improved the sensitivity of LGG cells to TMZ but also directly inhibited the proliferation and migration of LGG cells.

**Discussion:**

This study highlighted the association between peroxisomal FAO-related genes and poor prognosis in patients with LGG, and identified PMM2 as a candidate therapeutic target. Further studies investigating the role of peroxisomal FAO in glioma are urgently needed.

## Introduction

Metabolic reprogramming is one of the hallmarks of cancer. Metabolic reprogramming of tumors and the tumor microenvironment, including cells, nutrients, and metabolites, supports cancer proliferation, migration, and resistance (1, 2). In recent years, lipid metabolism reprogramming in cancer has attracted considerable attention. Fatty acids, as the basic molecules of lipids, play important roles in maintaining cell membrane homeostasis and regulating cell signal transduction and energy metabolism (3). Fatty acid (FA) chains have been shown to produce energy within growing tumors (4). Fatty acid β-oxidation is an important metabolic process for producing energy that occurs in both mitochondria and peroxisomes (5). The middle-chain FA is mainly catabolized in mitochondria, whereas the long-chain or very long-chain FA is mainly catabolized in peroxisomes (6). During tumor progression, cancer cells reprogram fatty acid oxidation (FAO) to produce ATP more efficiently. In particular, prostate cancer and breast cancer cells have been clearly demonstrated to utilize FAO as a metabolic strategy (7, 8). However, current studies of FAO in cancer have mainly focused on mitochondria, while research on peroxisomes remains limited.

Glioma is a highly aggressive and incurable disease. Low-grade glioma (LGG) is generally defined as World Health Organization (WHO) grade II and III glioma, with a relatively high 5-year survival rate, and mainly comprises IDH-mutant astrocytomas and oligodendrogliomas according to the WHO Classification of Tumors of the Central Nervous System (WHO CNS5, 2021) (9). However, although patients receive surgery combined with radiotherapy and temozolamide (TMZ), the majority of patients with LGG ultimately experience disease progression and recurrence, resulting in poor clinical outcomes (10–12). Therapeutic resistance and the strong proliferation and migration abilities of the tumor are considered the key causes of recurrence and progression. Recent research has emphasized that metabolic reprogramming is a key feature associated with chemoresistance in cancer cells. With respect to FAO in glioma, studies have demonstrated that FAO is required for the respiration and proliferation of malignant glioma, and the inhibition of FAO impairs ATP and NADPH production in pediatric glioma cell lines (13). However, studies exploring the functions of peroxisome-mediated FAO in LGG are lacking. How peroxisomal FAO-related genes affect LGG remains unclear.

This study utilized bioinformatics methods and functional assays to explore the potential roles of peroxisomal FAO-related genes in LGG. Our results demonstrated the promoting effect of peroxisomal FAO-related genes in LGG and identified PMM2 as a candidate gene associated with peroxisomal FAO that could serve as a potential therapeutic target for LGG. Further studies investigating the role of peroxisomal FAO in glioma are urgently needed.

## Methods

### Data acquisition and processing

The TCGA cohort consisted of 511 LGG samples from the TCGA database (https://portal.gdc.cancer.gov/). The CGGA1 cohort consisted of 479 LGG samples from the CGGA mRNAseq-693 dataset (http://www.genecards.org/). The CGGA2 cohort consisted of 172 LGG samples from the CGGA mRNAseq-325 dataset (http://www.cgga.org.cn/).

Samples without pathological information, grade information, and survival information were excluded. Genes with missing expression values in more than 75% of the samples were excluded. All RNA-sequencing data were converted to transcripts per million (TPM) formats and were log2 converted (value + 1) for subsequent analysis.

The peroxisomal FAO-related genes included in this study were selected based on their potential correlation, and 32 peroxisomal FAO-related genes were acquired from the GeneCards database (https://www.genecards.org/) (**Supplementary Table 1**). The GeneCards Inferred Functionality Score (GIFtS) algorithm uses the wealth of GeneCards annotations to produce annotation scores that predict the degree of a gene’s functionality. Because the degree of known function is related to the amount of research on a specific gene or its product, these annotation scores serve as inferred measures of function (14). GeneCards has been widely used for gene screening (15, 16). We used “peroxisome FAO” as the search keyword to find related genes and retained “protein coding” gens and “relevance score > 1.50” as peroxisomal FAO-related genes.

### Functional annotation of 32 peroxisomal FAO-related genes

KEGG and GO analyses were conducted to verify the functions of the 32 peroxisomal FAO-related genes acquired from the GeneCards database using the R “clusterProfiler” package. Pathways with a false discovery rate (FDR) < 0.25 and p < 0.05 were considered statistically significant and visualized using the “ggplot2” package.

### Clustering analysis

Cluster analysis was performed using Consensus Cluster Plus (17). An empirical cumulative distribution function plot was used to determine the optimal number of clusters.

### Construction and evaluation of the peroxisomal FAO-related genes prediction model

We integrated 10 machine learning algorithms and constructed 117 FAO-related genes prediction model using different algorithmic combinations. The model was constructed based on a previously published study (18). The detailed machine learning algorithms included: support vector machine (SVM), LASSO regression, gradient boosting machine (GBM), random forest, elastic net, stepwise Cox regression, Ridge, CoxBoost, super partial correlation (SuperPC), and partial least squares with Cox regression (plsRcox). The TCGA cohort was used as the training cohort, and the CGGA-1 and CGGA-2 cohorts were used as the validation cohorts. To avoid overfitting, we set 10-fold cross-validation when training the model (cv.folds = 10), and the validation cohorts were not used for iterative model selection. The reference source of the original code was obtained from https://github.com/l-magnificence/Mime. For each cohort, the Harrell’s concordance index (C-index) was calculated, and the average C-index of the two CGGA validation cohorts was defined as the mean C-index. The model with the highest mean C-index was selected as the optional model, indicating the greatest consistency across the validation cohorts and the highest robustness while minimizing the potential influence of training-set overfitting.

The risk score (RS) for each patient was calculated, and patients were grouped according to the median RS. Kaplan-Meier (KM) analysis was conducted to evaluate the difference in OS between the low- and high-RS subgroups using the R package “survival”. Time-dependent receiver operating characteristic (ROC) analysis was conducted to evaluate the prognostic efficacy of the FAO-related genes prediction model using the R package “survivalROC”.

### Multivariable COX regression analysis and nomogram construction

To assess the independent prognostic effects of the genes in the FAO-related genes prediction model, we conducted multivariate Cox proportional hazard regression analysis using the R package “survival”. The results were presented as hazard ratios (HR), 95% confidence intervals (95% CIs), and corresponding p values. A p-value of less than 0.05 was considered statistically significant.

The nomogram was the visualization and validation of the regression model and was constructed using the R package “rms”. By constructing a multivariate COX regression model, according to the contribution of each independent variable to the outcome, each value level of each independent variable was scored (points), and then each score was added to obtain the total score (total points). The precision of the projected survival rates at 1-, 3-, and 5-year OS was validated by generating calibration plots using the R package “pec”. The decision curve analysis (DCA) was performed using the R package “rmda v1.6”.

### GSEA analysis and temozolomide sensitivity evaluation

GSEA was used to explore enriched biological pathways in the high-RS subgroup using the R package “clusterProfiler”. The TMZ sensitivity of LGG samples was also predicted by estimating the half-maximum inhibitory concentration (IC_50_) using the Genomics of Drug Sensitivity in Cancer database and the R package “oncoPredict”.

### Cell culture

The two glioma cell lines, SW1088 and SW1783, and a normal glial cell line, HA, were obtained from Procell (Wuhan, China). Generally, SW1088 and SW1783 are widely used as validation cell lines for LGG (19–21). TMZ-resistant cells (SW1088-R and SW1783-R) were derived from SW1088 and SW1783 cells. According to previous studies, TMZ-resistant cell lines were established by continuous stepwise selection using increasing concentrations of TMZ (22, 23). Specifically, TMZ-resistant LGG cell lines were derived from the parental cell lines by treatment with gradually increasing concentrations of TMZ (6.25, 12.5, 25, 50, and 100 μM). The TMZ-resistant LGG cell lines were generated after 6 months of chronic exposure to TMZ and were maintained in a clinical dose (100 μM) of TMZ. All cells were maintained at 37°C with 100% humidity and 5% CO_2_. All cells were cultured in Dulbecco’s Modified Eagle Medium with 10% fetal bovine serum and 1% penicillin/streptomycin.

### Lentiviral transfection

Lentiviruses (GeneChem, Shanghai, China) with the following sequences were used: sh1#1: ATGTTAAACGTGTCCCCTAT, sh2#2: CTTTCATTGAATTCCGAAAT. After optimization according to the manufacturer’s protocol, a multiplicity of infection (MOI) of 5 was selected for cell transfection. The cells were cultured under conventional conditions, and cells in a good growth state were selected for lentiviral transduction. The cells were cultured at 37°C and 5% CO_2_ for 24 h after the addition of virus-containing medium. After 24 h, the virus-free medium was replaced to continue incubation. Puromycin (2μg/mL) was used to screen stable strains for 3 weeks. Finally, quantitative real-time PCR (qRT-PCR) was performed to test the knockdown efficiency. All procedures complied with Biosafety Level 2 guidelines.

### RNA extraction, cDNA synthesis, and quantitative real-time PCR

AG RNAex Pro reagent (Accurate Biology, #cat: AG21102) was utilized to extract total RNA from cell lines based on the reagent instructions. The SYBR Green SupTaq HS premix qRT-PCR kit (Accurate Biology, AG11762) was used for gDNA removal, cDNA synthesis, and PCR amplification. After the completion of the preparation of the reaction system, the amplification was performed using the ABI QuantStudio 1 Plus real-time fluorescence quantitative PCR system. Primer sequences for qRT-PCR were used: GAPDH, F: 5’-GGTGGTCTCCTCTGACTTCAAC-3’, R: 5’-GTTGCTGTAGCCAAATTCGTTGT-3’; PMM2, F: 5’-TCCAGAAAATGGCTTGGTAGC-3’, R: 5’-ACCCCTCTTCTTCGGGAGTT-3’. The 2−ΔΔCt method was used to process qPCR data to obtain the relative expression of genes.

### Evaluation of IC_50_

The IC_50_ values of LGG cells were determined using the Cell Counting Kit-8 (CCK8). All LGG cell lines were seeded at a density of 5 × 10^3^/well and treated with the same concentrations of TMZ. After treatment for 48 h, 10 μL of CCK-8 reagent was added to each well, and the absorbance was analyzed at 450 nm using a microplate reader (PerkinElmer), with wells without cells serving as blanks. The IC_50_ values of the cells were presented using dose-response curves generated using Prism 9.0 software.

### EdU incorporation assay

The proliferation of LGG cell lines was assessed using the EdU Cell Proliferation Kit (BeyoClick™, China). LGG cells were seeded in 24-well plates and treated with 10 μM EdU for 2 h at 37 °C. After fixation with 4% paraformaldehyde for 15 min and permeabilization with 0.3% Triton X-100 for 20 min, cells were stained with Click Reaction Mix (containing Alexa Fluor 594) for 30 min in the dark. Nuclei were labeled with Hoechst 33,342 (Beyotime, China) for 10 min. Fluorescent images were acquired using a Nikon Eclipse Ti2 microscope (Japan), and EdU-positive cells were presented using ImageJ software (NIH, United States).

### Transwell migration assay

The LGG cells were seeded in the upper chamber of Transwell inserts containing 200 µL of serum-free medium, while the lower chamber contained 500 µL of medium supplemented with 20% fetal bovine serum. After 24 h of incubation at 37°C, cells that migrated to the lower chamber were fixed with 4% paraformaldehyde, stained with 0.1% crystal violet, and visualized under an optical microscope.

### Data analysis

The software R Studio (version 4.2.3) and GraphPad Prism 9.0 (San Diego, CA) were used for bioinformatics analyses and visualizations. All experimental statistical analyses were performed using between-group differences and were analyzed using one-way ANOVA with Sidak’s multiple-comparisons test or the Wilcoxon test (p < 0.05). Pearson’s correlation coefficient was used for correlation analysis. Data are presented as mean ± standard deviation (SD) unless otherwise specified. Significance levels were: *P<0.05, **P< 0.01, ***P< 0.001, and ****P<0.0001. A significance level of p < 0.05 was considered statistically significant.

## Result

### Peroxisomal FAO-related genes affect the prognosis of LGG

**Figure 1:**
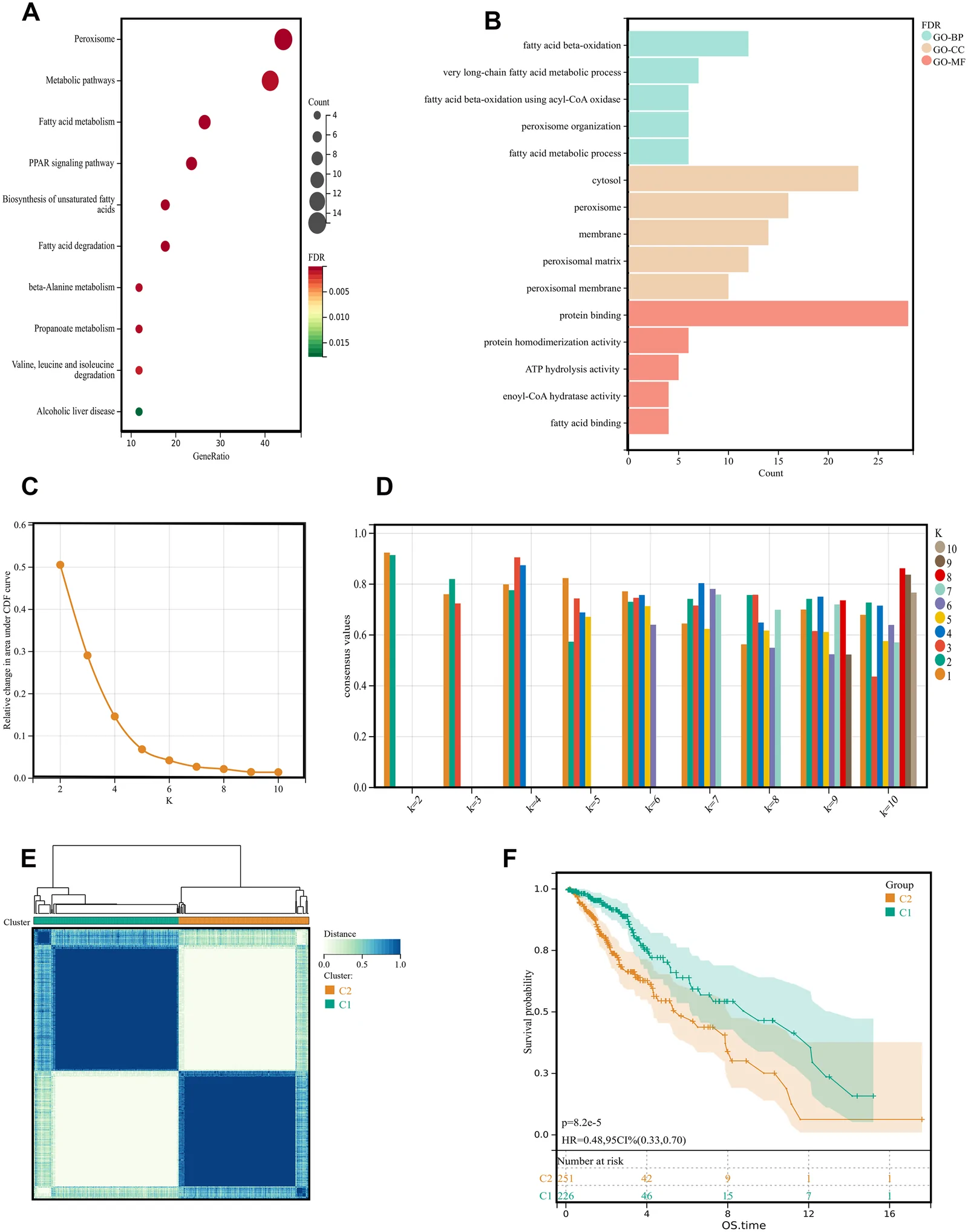
**(A)** KEGG enrichment analysis of the 32 peroxisome FA beta-oxidation-related genes; **(B)** GO enrichment analysis of the 32 peroxisome FA beta-oxidation-related genes; **(C)** The CDF curve of the clustering analysis; **(D)** The setting of K value and the corresponding consensus value; **(E)** The optimal clustering result; **(F)** The Kaplan-Meier analysis comparing the C1 and C2 clusters.

We extracted the expression matrix of 32 peroxisomal FA beta-oxidation-related genes and conducted KEGG and GO analyses to validate their functions. The function annotation results displayed that the 32 genes widely participated in peroxisomal FA beta- oxidation-related processes (**Figures 1A, B**). Then, consensus clustering analysis based on the expressions of the 32 genes in LGG samples in the TCGA dataset was performed based upon K-Medoids algorithm.. According to the results of the area under the CDF curve and the intra-group sample consistency evaluation, when K = 2, the intra-group sample consistency reached the highest level and the optimal clustering performance (**Figures 1C, D**). The cluster heatmap demonstrated that the expression levels of these 32 genes were significantly different between the clusters (**Figure 1E**). To explore whether these 32 genes affected the survival of LGG patients, we conducted K-M analysis. The results demonstrated that patients in cluster C2 had a poorer prognosis than those in cluster C1, suggesting that peroxisomal FAO-related genes influence the prognosis of LGG (**Figure 1F**).

### Construction of the peroxisomal FAO-related prognostic model

**Figure 2:**
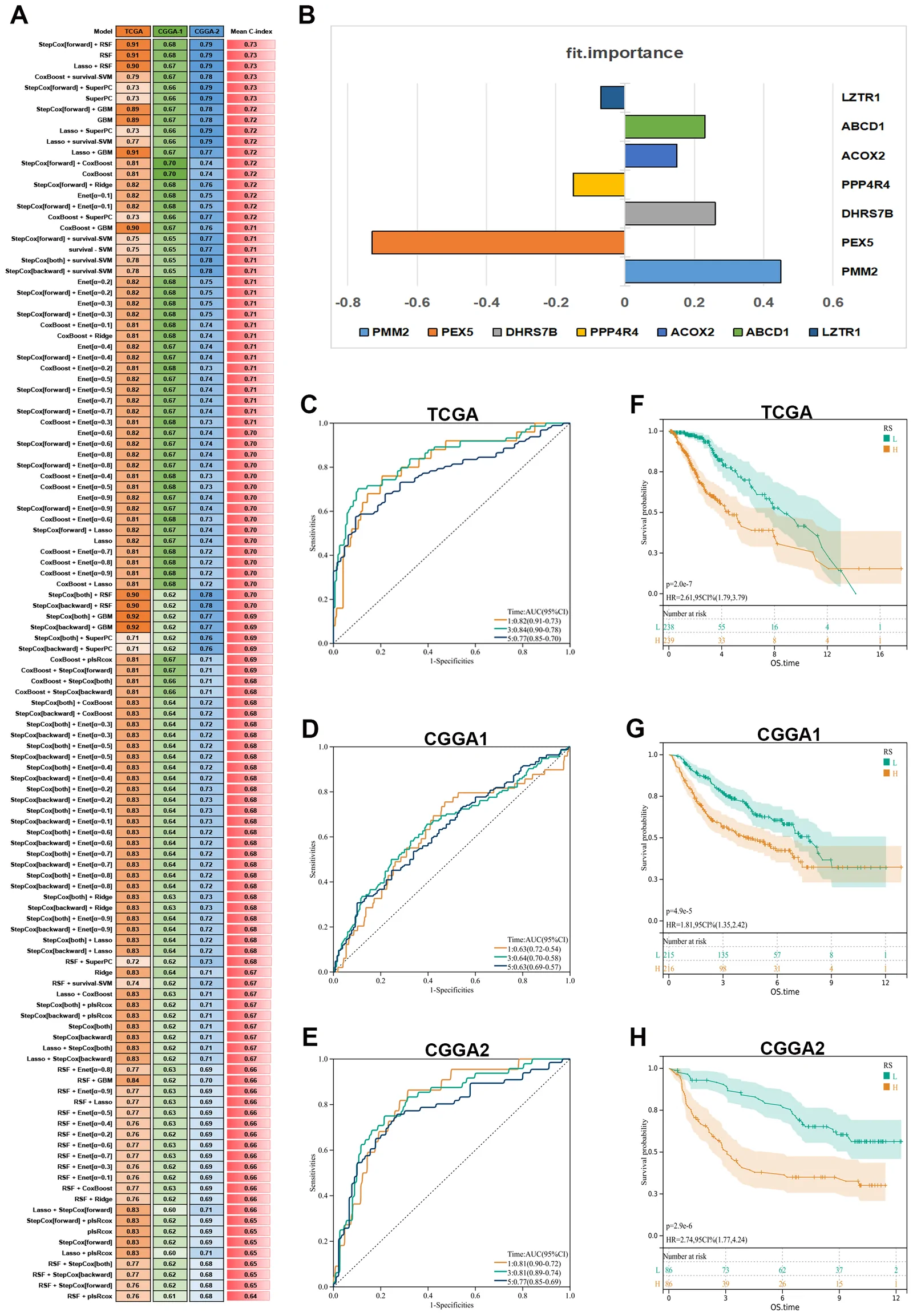
**(A)** Construction of peroxisomal FAO-related prognostic model through machine learning algorithms; **(B)** Features and their corresponding importance in the optimal model; **(C-E)** ROC curve analyses of 1-, 3-, and 5-years OS in the TCGA, CGGA1, and CGGA2 cohorts, respectively; **(F-H)** Kaplan-Meier survival analysis of OS between high- and low-RS subgroups in the TCGA, CGGA1, and CGGA2 cohorts.

To identify the prognostic peroxisomal FAO-related genes, we utilized the combinations of 10 well-established machine learning algorithms to develop a robust peroxisomal FAO-related prognostic model. By comparing the mean C-index in each combined model, the model based on the combination of StepCox [forward] and RSF achieved the highest mean C-index, indicating the greatest predictive consistency across the training cohort and the validation cohorts (**Figure 2A**). There were 7 peroxisomal FAO-related genes included in the model: PMM2, PEX5, DHRS7B, PPP4R4, ACOX2, ABCD1, and LZTR1. The RS was calculated as follows:

RS = 0.45*PMM2 - 0.73*PEX5 + 0.26*DHRS7B - 0.15*PPP4R4 + 0.15*ACOX2 + 0.23*ABCD1 - 0.07*LZTR1, which was the quantification of the model (**Figure 2B**).

To examine the predictive value of the peroxisomal FAO-related prognostic model, we conducted K-M analysis and time-dependent ROC analyses in the training and validation cohorts. The AUC values in the TCGA training cohort were 0.82 (0.73–0.91) for 1-year OS, 0.84 (0.78–0.90) for 3-year OS, and 0.77 (0.70–0.85) for 5-year OS. The AUC values in the CGGA1 validation cohort were 0.63 (0.54–0.72) for 1-year OS, 0.64 (0.58–0.70) for 3-year OS, and 0.63 (0.57–0.69) for 5-year OS. The AUC values in the CGGA2 validation cohort were 0.81 (0.72–0.90) for 1-year OS, 0.81 (0.74–0.89) for 3-year OS, and 0.77 (0.69–0.85) for 5-year OS (**Figures 2C–E**). These results supported the reliable survival prediction value of the peroxisomal FAO-related prognostic model. K-M analysis demonstrated that the peroxisomal FAO-related prognostic model can effectively stratify LGG patients according to prognosis. High-RS patients had poorer prognosis than low-RS patients, and this finding was consistent across all the cohorts (**Figures 2F–H**).

### The clinical importance of the peroxisomal FAO-related prognostic model

To further evaluate the clinical importance of the peroxisomal FAO-related prognostic model, we conducted multivariable Cox regression analysis, which employed the peroxisomal FAO-related prognostic model and various identified clinical prognostic variables, including WHO grade, IDH mutation status, 1p/19q co-deletion status, and MGMT methylated status. The results of the multivariable Cox regression analysis suggested that the prognostic effect of the peroxisomal FAO-related prognostic model was independent of the above mentioned molecular classifications (**Supplementary Figures 2A–C**, **Supplementary Table 3**).

**Figure 3:**
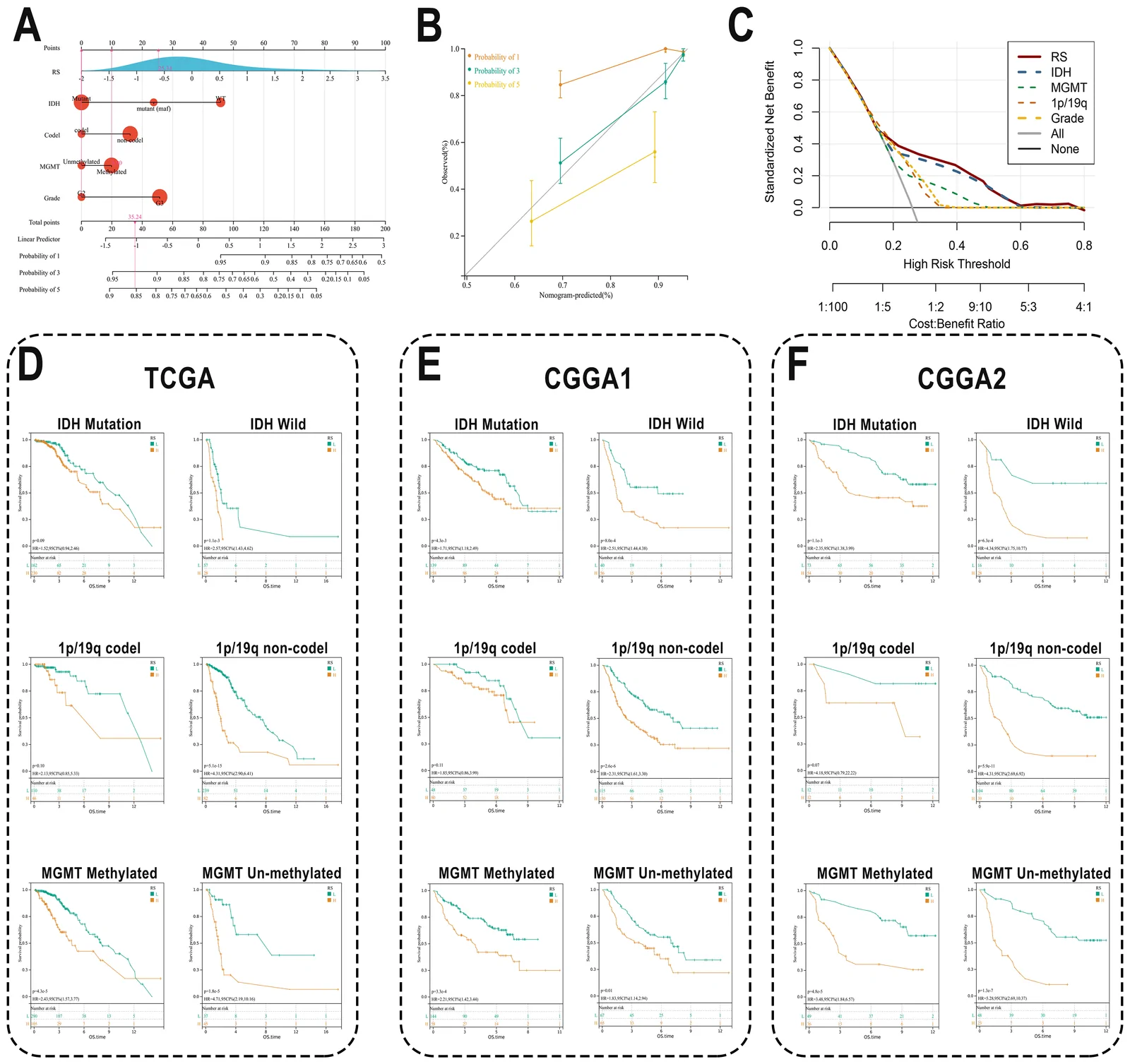
**(A)** Nomogram for predicting survival using glioma clinical prognostic factors and peroxisomal FAO-related prognostic model; **(B)** Calibration curve for nomogram; **(C)** Decision Curve Analysis (DCA) suggested that the net benefit of the predictive strategy based on the peroxisomal FAO-related prognostic model significantly exceeded that of the predictive strategy based on the known clinical prognostic variables; **(D)** The stratification effects of RS in LGG patients with different molecular status in the TCGA cohort; **(E)** The stratification effects of RS in LGG patients with different molecular status in the CGGA1 cohort; **(F)** The stratification effects of RS in LGG patients with different molecular status in the CCGA2 cohort. Statistical significance was determined by the log-rank test. P<0.05 was considered to indicate statistical significance.

Moreover, we constructed a nomogram based on the multivariable Cox regression analysis. The length of the corresponding line segment of each variable in the nomogram represented the strength of the influence of each variable on the outcome. The peroxisomal FAO-related prognostic model exerted a stronger influence on the OS of LGG patients than the established clinical prognostic variables (**Figure 3A**). The calibration plot indicated that the constructed nomogram accurately predicted the 1-, 3-, and 5-year survival probabilities (**Figure 3B**). In addition, the DCA demonstrated substantial positive net benefits across nearly all threshold probabilities at different time points, suggesting that the peroxisomal FAO-related prognostic model has good potential clinical efficacy (**Figure 3C**). Based on these findings, we evaluated the prognostic value of the peroxisomal FAO-related prognostic model across different LGG subtypes. The results suggested that the peroxisomal FAO-related prognostic model can further stratify patients according to prognosis, exhibiting higher prognostic value than the existing clinical biomarkers (**Figures 3D–F**). Notably, even in IDH-mutant combined with 1p/19q-codeleted glioma patients and IDH-mutant combined with 1p/19q-non-codeleted glioma patients, the peroxisomal FAO-related prognostic model successfully stratified patients with different prognosis (**Supplementary Figures 2D–F**).

**Figure 4:**
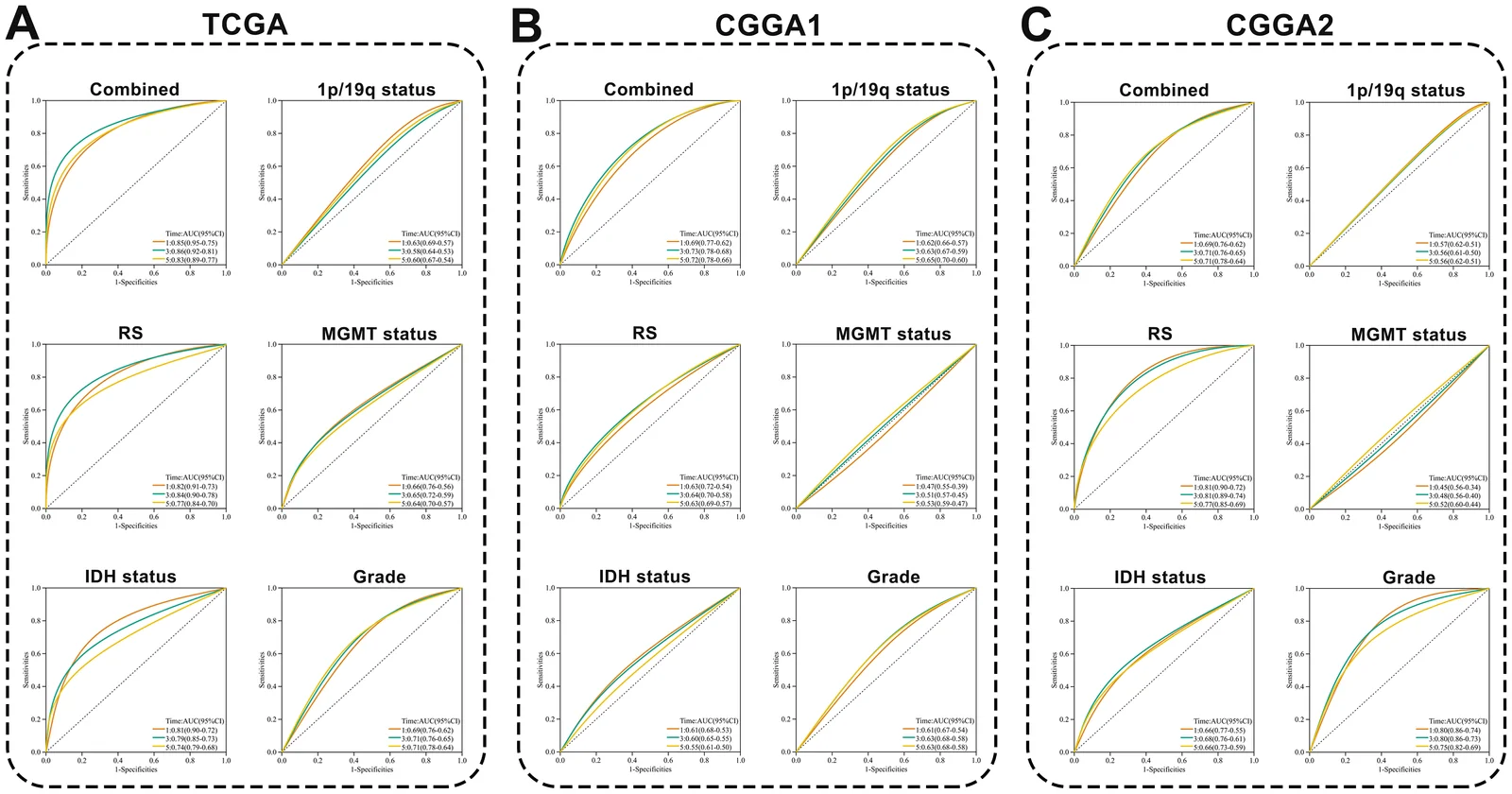
**(A)** ROC curve analyses of 1-, 3-, and 5-years OS for RS, IDH status, 1p/19q status, MGMT status, Grade, and combining all variables in the TCGA cohort; **(B)** The ROC curve analysis of 1-, 3-, and 5-years OS for RS, IDH status, 1p/19q status, MGMT status, Grade, and combining all variables in the CCGA1 cohort; **(C)** The ROC curve analysis of 1-, 3-, and 5-years OS for RS, IDH status, 1p/19q status, MGMT status, Grade, and combining all variables in the CCGA2 cohort.

To compare the prognostic value of the peroxisomal FAO-related prognostic model with that of established clinical prognostic variables more directly, we conducted time-dependent ROC analyses based on 1-, 3-, and 5-year OS. Across all cohorts, the AUC values for 1-, 3-, and 5-year OS of the peroxisomal FAO-related prognostic model consistently exceeded those of the established clinical variables. Furthermore, combining our peroxisomal FAO-related prognostic model with the clinical variables yielded the highest AUC values for 1-, 3-, and 5-year OS, suggesting that the combined model can partly compensate for the limitations of each individual variable (**Figures 4A–C**).

### The peroxisomal FAO-related prognostic model predicted the TMZ sensitivity

**Figure 5:**
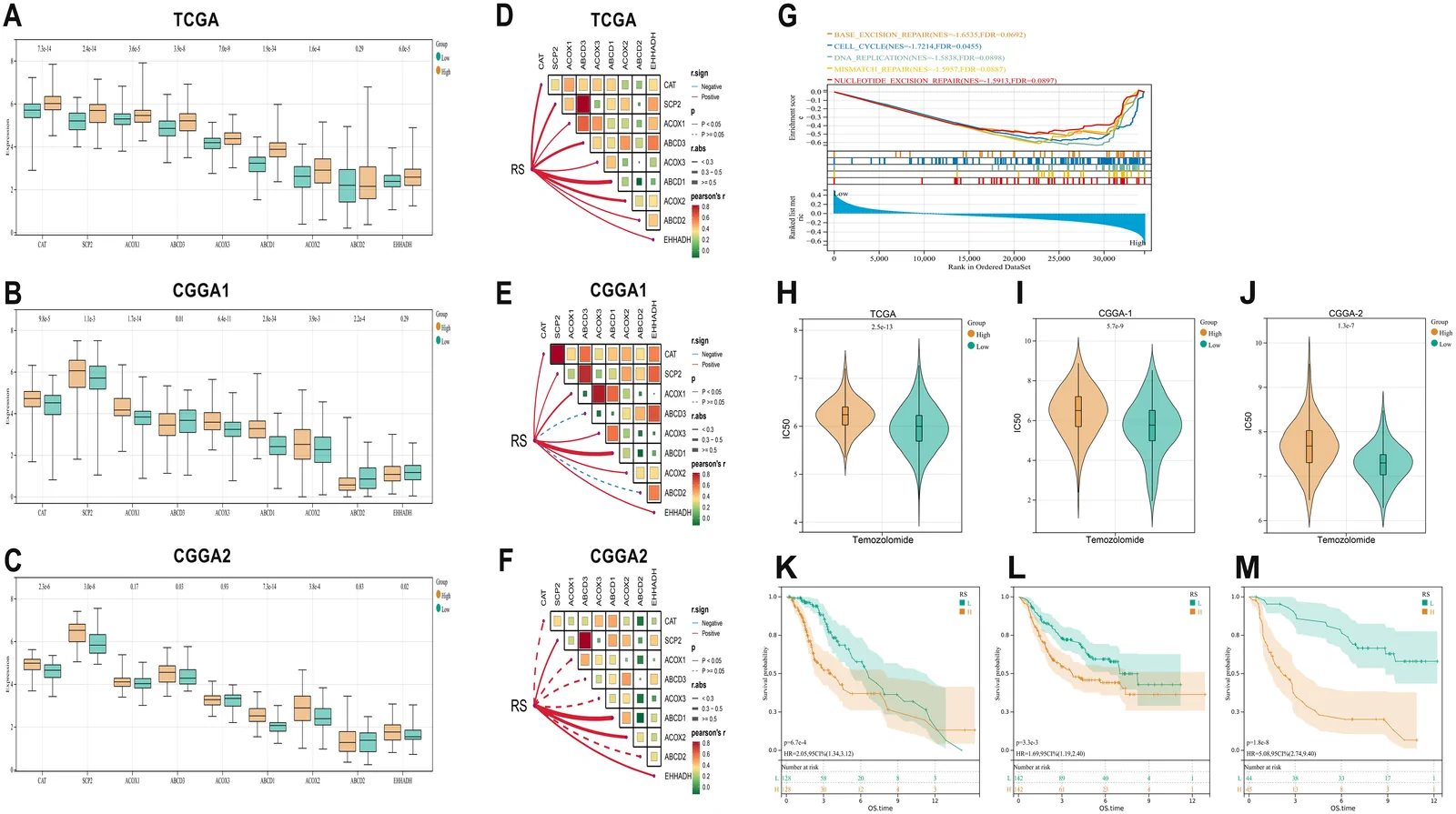
**(A-C)** The expression levels of key enzymes and markers of the peroxisome in the high- and low-RS subgroups in the TCGA, CGGA1, and CGGA2 cohorts; **(D-F)** The correlations between RS and the expression levels of key enzymes and markers of the peroxisome in the TCGA, CGGA1, and CGGA2 cohorts. The color of the lines between RS and the heat map represents the correlation with the corresponding gene expression (red = positive, blue = negative). The type of line segment represents the statistical significance of the correlation between RS and corresponding gene expression (dashed line = p>0.05, solid line = p<0.05). The thickness of the line segment represents the strength of the correlation between RS and the corresponding gene expression (the thicker the line segment, the stronger the correlation). **(G)** GSEA analysis revealed the pathways enriched in the high-RS subgroup. **(H-J)** The IC_50_ of samples of TMZ between the high- and low-RS subgroups in the TCGA, CGGA1, and CGGA2 cohorts; **(K-M)** The stratification effects of RS in LGG patients who received TMZ treatment in the TCGA, CGGA1, and CGGA2 cohorts. Statistical significance was determined by Wilcoxon test. Pearson’s coefficient was used for correlation analysis. Statistical significance of K-M analysis was determined by the log-rank test. P< 0.05 was considered statistically significant (*p < 0.05; **p < 0.01; ***p < 0.001, ****p < 0.0001).

To reflect differences in peroxisome-mediated FAO level between the low- and high-RS LGG subgroups, we compared the expression levels of key enzymes in peroxisomal FAO and key peroxisomal markers between the two subgroups. Notably, the majority of the key peroxisomal FAO enzymes, such as ACOX1, ACOX2, and SCP2, were significantly upregulated in high-RS subgroups in all the cohorts. Similarly, the expression levels of key peroxisomal markers, such as CAT and ABCD1, were also upregulated in the high-RS subgroups in every cohort (**Figures 5A–C**). Correlation heatmaps further demonstrated that the RS was positively correlated with the expression of the majority of key enzymes in peroxisomal FAO and key peroxisomal markers across all cohorts (**Figures 5D–F**). These results further validate that the prognosis of LGG patients was affected by peroxisomal FAO.

To investigate how the prognostic peroxisomal FAO-related genes influenced the survival of LGG patients, we performed GSEA analysis to identify potential mechanisms. Our results indicated that the majority of the non-specific DNA damage repair pathways were enriched in the high-RS group, suggesting that the therapy resistance may be the key mechanism (**Figure 5G**). Considering that activation of non-specific DNA damage repair pathways can reverse the damage of TMZ to glioma and that lipid metabolic reprogramming contributes to the treatment resistance of various cancers, we performed drug sensitivity prediction analyses. Results across all cohorts consistently exhibited that the predictive IC_50_ values of TMZ in the high-RS subgroup were higher than in the low-RS subgroup (**Figures 5H–J**). We then conducted K-M analysis to evaluate the prognostic effect of the peroxisomal FAO-related prognostic model. The RS was positively correlated with the poor prognosis of LGG patients who received TMZ therapy, indicating that the high-RS subgroup may represent a TMZ resistance-associated phenotype (**Figures 5K–M**).

### Identification and validation of PMM2 as a prognostic and therapeutic marker for LGG

**Figure 6:**
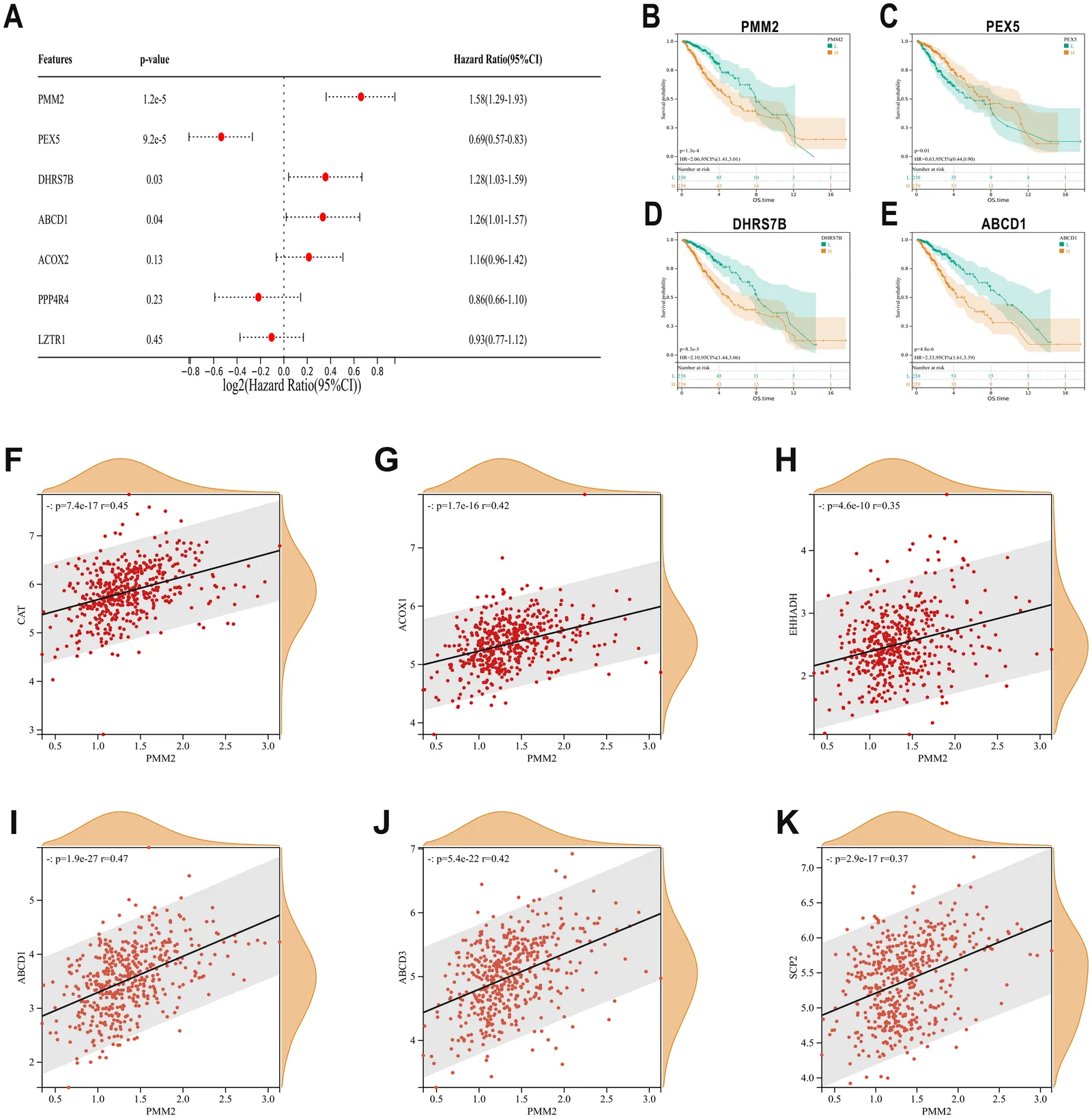
**(A)** Mutivariable Cox regression analysis evaluating the independent prognostic effects of the features in the model; **(B)** Kaplan-Meier survival analysis of OS between patients with high and low expression of PMM2; **(C)** Kaplan-Meier survival analysis of OS between patients with high and low expression of PEX5; **(D)** Kaplan-Meier survival analysis of OS between patients with high and low expression of DHRS7B; **(E)** Kaplan-Meier survival analysis of OS between patients with high and low expression of ABCD1; **(F)** The correlation between the expression of PMM2 and CAT; **(G)** The correlation between the expression of PMM2 and ACOX1; **(H)** The correlation between the expression of PMM2 and EHHADH; **(I)** The correlation between the expression of PMM2 and ABCD1; **(J)** The correlation between the expression of PMM2 and ABCD3; **(K)** The correlation between the expression of PMM2 and SCP2. Pearson’s coefficient was used for correlation analysis. P < 0.05 was considered to indicate statistical significance (*p < 0.05; **p < 0.01; ***p < 0.001, ****p < 0.0001).

Multivariable Cox regression analysis was performed to identify the independent prognostic peroxisomal FAO-related genes among the seven genes included in the model. The results demonstrated that PMM2, PEX5, DHRS7B, and ABCD1 were independent prognostic factors. Among these genes, PMM2, DHRS7B, and ABCD1 were identified as risk factors for prognosis, while PEX5 served as a protective factor (**Figure 6A**). K-M analysis further validated that PMM2, PEX5, DHRS7B, and ABCD1 can independently stratify LGG patients with different prognoses. Patients with high expression levels of PMM2, DHRS7B, and ABCD1 had a poorer prognosis, while patients with high PEX5 expression had a better prognosis (**Figures 6B–E**). Among these genes, PMM2 demonstrated the strongest independent prognostic value. Correlation analysis further revealed that the expression of PMM2 was positively correlated with several hub genes involved in the peroxisomal FAO process, including CAT (r = 0.45, p = 7.4e-17), ACOX1 (r = 0.42, p = 1.7e-16), EHHADH (r = 0.35, p = 4.6e-10), ABCD1 (r = 0.47, p = 1.9e-27), ABCD3 (r = 0.42, p = 5.4e-22), and SCP2 (r = 0.37, p = 2.9e-17) (**Figures 6F–K**), indicating that PMM2 may represent a novel candidate gene associated with peroxisomal FAO.

**Figure 7:**
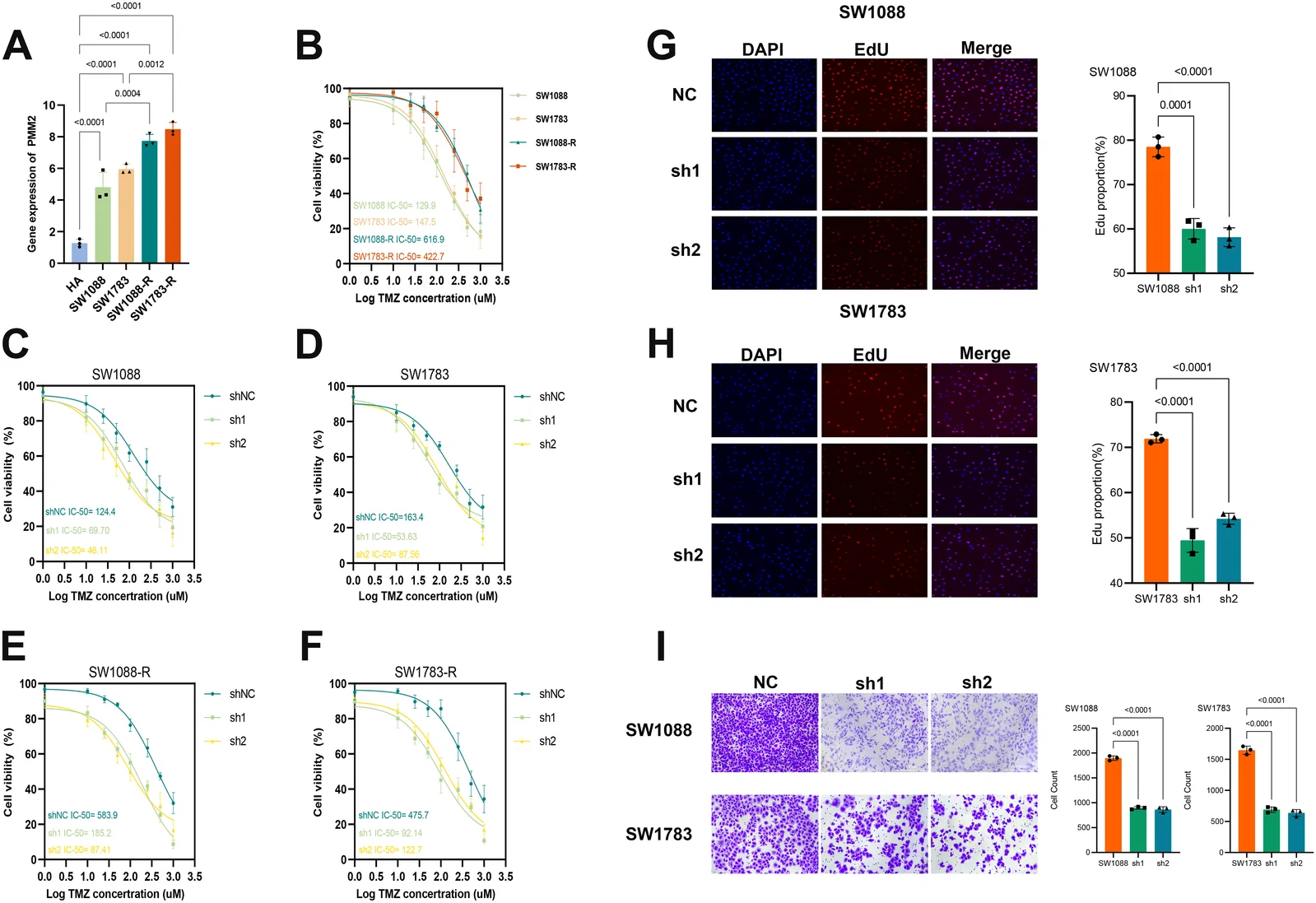
**(A)** The expression level of PMM2 in different cell lines based on qRT-PCR (n = 3); **(B)** The baseline IC50 of TMZ of different LGG cell lines presented via cell survival (n = 3); **(C)** The knockdown of PMM2 impeded the resistance to TMZ in SW1088 cells (n = 3); **(D)** The knockdown of PMM2 impeded the resistance to TMZ in SW1783 cells (n = 3); **(E)** The knockdown of PMM2 impeded the resistance to TMZ in SW1088-R cells (n = 3); **(F)** The knockdown of PMM2 impeded the resistance to TMZ in SW1783-R (n = 3); **(G)** EdU assay suggested the downregulation of PMM2 impeded the proliferation of SW1088 cell line (n = 3); **(H)** EdU assay suggested the downregulation of PMM2 impeded the proliferation of SW1783 cell line (n = 3); **(I)** Transwell assay suggested the downregulation of PMM2 in SW1088 and SW1783 cell lines impeded their migration ability(n=3). Tukey’s multiple comparisons test was used to examine the differences in PMM2 expression among different cell lines. P < 0.05 was considered to indicate statistical significance (*p < 0.05; **p < 0.01; ***p < 0.001, ****p < 0.0001).

Considering that PMM2 exhibited the strongest independent prognostic effect and that little research has investigated its biological function in glioma, we performed *in vitro* experiments to explore its function in LGG cell lines. Because the previous analysis suggested that the high-RS subgroup had stronger TMZ resistance characteristics, we constructed TMZ-resistant LGG cell lines and examined the expression level of PMM2 in these cells. The result of qRT-PCR demonstrated that PMM2 was upregulated in LGG cell lines compared with normal glial cells (HA), and its expression was significantly higher in TMZ-resistant LGG cell lines (**Figure 7A**). The IC_50_ values of TMZ-resistant LGG cells (SW1783-R: 422.7 μM, SW1088-R: 616.9 μM) were significantly increased compared with those of the parental LGG cells (SW1783: 147.5μM, SW1088: 129.9 μM) (**Figure 7B**).

We next performed PMM2 knockdown experiments to further explore its function in TMZ resistance (**Supplementary Figure 3**). Results revealed that the downregulation of PMM2 significantly decreased the IC_50_ of TMZ in both the TMZ-resistant LGG cell lines and the routine LGG cell lines (**Figures 7C–F**), indicating that the inhibition of PMM2 improved their sensitivity to TMZ.

As for functional assays, the result of the EdU incorporation assay revealed that the inhibition of PMM2 impeded the proliferation of the LGG cell lines (**Figures 7G, H**). The Transwell assay revealed that the downregulation of shPMM2 impaired cell migration (**Figure 7I**). Based on the above, it can be inferred that PMM2, on the one hand, contributed to TMZ resistance of LGG cells and, on the other hand, directly contributed to the proliferation and migration of LGG, restricting the OS of patients with LGG.

## Discussion

Exploring the biological mechanisms of treatment resistance and recurrence remains a major focus of current research on glioma. In recent years, increasing evidences has suggested that metabolic reprogramming during treatment may be one of the key reasons for the failure of cancer treatment. An increasing number of studies have reported that lipid metabolism disorders contributes to progression and therapeutic resistance in various cancers (24, 25). Fatty acids, as an important energy source, can provide energy for tumor cells through FAO, thereby supporting their survival, invasion, metastasis, and drug resistance, especially under stress conditions such as nutrient deficiency or hypoxia.

FAO occurs not only in mitochondria but also in peroxisomes (5). At present, the majority of studies have focused on the effects of mitochondrial FAO on the malignant characteristics of cancer, while relatively little attention has been paid to peroxisomes, the ones responsible for oxidation. In this study, we first used GeneCards to screen genes that have potential correlations with the peroxisomal FAO process by setting a threshold. To verify the function of these genes, we then performed pathway analysis (KEGG/GO) on these genes and found that these genes were widely enriched in the “peroxisomal fatty acid oxidation” related pathway. GeneCards and the curated pathway resources (KEGG/GO) complement each other because many biological processes are directly or indirectly influenced by multiple genes, although the roles of some genes have not yet been experimentally established. GeneCards also provide predicted gene functionality (14), helping us identify novel genes that may participate in the peroxisomal FAO process. As a result, we included 32 peroxisomal FAO-related genes for subsequent analyses.

Based on the expression profiles of 32 peroxisomal FAO-related genes, LGG samples were divided into two subtypes with significant survival differences by consensus cluster analysis, suggesting that peroxisomal FAO-related genes may influence the prognosis of patients with LGG. To further identify potential prognostic biomarkers, we constructed a robust peroxisomal FAO-related prognostic model using 10 machine learning algorithms. The model consisted of seven genes: PMM2, PEX5, DHRS7B, PPP4R4, ACOX2, ABCD1, and LZTR1. The model demonstrated excellent prognostic efficacy in both the training and validation cohorts based on ROC analysis, and the K-M curves revealed that patients with high-RS generally had a poorer prognosis than those with low-RS. Furthermore, compared with established clinical prognostic variables, such as IDH status, 1p/19q status, MGMT status, and grade, our peroxisomal FAO-related prognostic model achieved higher AUC values for 1-, 3-, and 5-year OS. Combining our model with these clinical variables produced the best performance. These results further demonstrate the strong clinical prognostic value of our peroxisomal FAO-related prognostic model, suggesting it may partly compensate for the limitations of established variables and have a potential clinical translation value.

Moreover, the expression of peroxisomal markers (CAT and ABCD1) and key enzymes of peroxisome-mediated FAO (ACOX1, ACOX2, and SCP2) were consistently upregulated in the high-RS subgroup, suggesting that the increased number of peroxisomes and the enhanced function of the peroxisomal FAO process contribute to the poorer prognosis of LGG. Meanwhile, an increasing number of studies have emphasized the promoting roles of peroxisomes in tumor progression. It was reported that targeted inhibition of PEX3 or PEX19 can weaken peroxisome biosynthesis and make melanoma sensitive to MAPK pathway inhibitors, thereby overcoming drug resistance (26). In prostate cancer, the inhibition of PPAR-α reduced the peroxisome-derived ROS, which suppressed cell proliferation and increased cellular sensitivity to the antiandrogen enzalutamide in prostate cancer cells (27). These results collectively indicate that peroxisomes play an important role in supporting tumor therapeutic resistance and malignant phenotypes. Similarly, our analysis found that multiple non-specific DNA damage repair pathways, such as “base excision repair” and “nucleotide excision repair”, were significantly enriched in the high-RS subgroup, and the predictive half maximal inhibitory concentration (IC_50_) of TMZ was also higher in the high-RS subgroup, suggesting that peroxisomal FAO-related prognostic signature is closely correlated with the TMZ resistance-associated phenotype. Previous studies have also reported that the expression of some molecules that can predict TMZ response in patients with glioma were significantly correlated with the enrichment of DNA damage repair pathways (28). It is worth noting that temozolomide (TMZ), a basic drug for glioma chemotherapy, develops therapeutic resistance that is strongly associated with the activation of non-specific DNA damage repair pathways. The activation of these pathways can clear O-6-methylguanine adducts and restore genomic integrity, thereby repairing the damage caused by TMZ (29).

Finally, multivariable Cox regression analysis showed that PMM2, PEX5, DHRS7B, and ABCD1 were independent prognostic factors for LGG, with PMM2 demonstrating the strongest independent prognostic effect. Previous studies on PMM2 have shown that its abnormal expression is associated with the progression of various cancers, including breast cancer, renal cell carcinoma, and colorectal cancer (30–32). However, PMM2 is not a canonical peroxisomal FAO gene. Therefore, we conducted correlation analysis to further evaluate whether PMM2 could represent a potential peroxisomal FAO-related gene. Our analysis indicated that the expression of PMM2 was significantly positively correlated with CAT, ACOX1, EHHADH, ABCD1, ABCD3, and SCP2. CAT is a marker enzyme of the peroxisome (33). ACOX1, EHHADH, and SCP2 are key enzymes of the peroxisomal fatty acid beta-oxidation, with ACOX1 serving as the first rate-limiting enzyme (34–36). The final step of the peroxisomal beta-oxidation pathway is catalyzed by sterol carrier protein-x (SCPx), which is encoded by the SCP2 gene (36). ABCD1 and ABCD3 are located in the peroxisomal membrane and are responsible for the transport of various lipid substrates into the peroxisome for their shortening by beta-oxidation (37). Therefore, our analysis further indicated that PMM2 may be a novel candidate gene correlated with the peroxisomal FAO process. To date, few studies have investigated the biological functions of PMM2 in glioma. To address this knowledge gap, *in vitro* experiments were conducted. Our study found that the expression of PMM2 not only contributed to TMZ resistance in LGG cells but also directly regulated their malignant behaviors. Our results indicated that PMM2 may be a potential key metabolic oncogene in LGG and a potential clinical intervention target.

### Limitation

Although our results preliminarily demonstrated that the peroxisomal FAO-related prognostic model we established had a relatively high predictive effect, there are several limitations that should be acknowledged. The AUC values from the time-dependent ROC analysis and the C-index in the CGGA1 cohort were downregulated compared with the other cohorts, indicating a certain degree of performance heterogeneity. This finding highlights the need for more cohorts to expand the sample size to further improve the stability and generalizability of our model. Furthermore, as noted above, the peroxisomal FAO-related genes in this study were selected based on their potential correlation, and several genes in our model, including PMM2, are not canonical peroxisomal FAO genes. Therefore, further mechanistic studies and *in vivo* validations are needed to explore the correlations between these genes, particularly the key prognostic gene PMM2, and the peroxisomal FAO process

## Conclusion

In summary, this study focused on peroxisomal FAO-related genes, constructing a reliable prognostic model based on machine learning. Our study preliminarily uncovered that the peroxisomal FAO process may have an adverse impact on the prognosis of LGG and identified PMM2 as a novel candidate gene associated with the peroxisomal FAO. Targeting PMM2 may represent a potential new therapeutic strategy for LGG treatment. Considering that the existing research on the exploration of peroxisomes in glioma remains limited, further studies investigating peroxisomal FAO-related genes in glioma are warranted.

## Data Availability

The original contributions presented in the study are included in the article/**Supplementary Material**. Further inquiries can be directed to the corresponding author.

## Supplementary material

Supplementary Figure 1 The workflow for machine learning.

Supplementary Figure 2 **(A–C)** The visualization of multivariable COX regression analysis in TCGA, CGGA1, and CGGA2 cohorts, respectively. **(D–F)** K-M analysis demonstrated the stratification effect of the peroxisome FAO-related prognostic model on IDH-mutant+1p/19q-codeletion subtype gliomas and IDH-mutant+1p/19q-non-codeletion subtype gliomas in TCGA, CGGA1, and CGGA2 cohorts, respectively.

Supplementary Figure 3 **(A)** The knockdown efficiency of PMM2 in SW1088 cell line. **(B)** The knockdown efficiency of PMM2 in SW1088-R cell line. **(C)** The knockdown efficiency of PMM2 in SW1783 cell line. **(D)** The knockdown efficiency of PMM2 in SW1783-R cell line.

Supplementary Table 1 The full gene list with scores that obtained from GeneCards.

Supplementary Table 2 The 95% confidence intervals for C-index in each model.

Supplementary Table 3 The detailed multivariable Cox results for the RS alongside major clinical covariates in each cohort.
